# GRP78 enhances the glutamine metabolism to support cell survival from glucose deficiency by modulating the β-catenin signaling

**DOI:** 10.18632/oncotarget.2105

**Published:** 2014-06-14

**Authors:** Zongwei Li, Yingying Wang, Haili Wu, Lichao Zhang, Peng Yang, Zhuoyu Li

**Affiliations:** ^1^ Institute of Biotechnology, Key Laboratory of Chemical Biology and Molecular Engineering of National Ministry of Education, Shanxi University, Taiyuan, China; ^2^ College of Life Science, Zhejiang Chinese Medical University, Hangzhou, China

**Keywords:** GRP78, β-catenin, adenomatous polyposis coli, glucose metabolism, glutamine metabolism

## Abstract

To support the high rates of proliferation, cancer cells undergo the metabolic reprogramming: aerobic glycolysis and glutamine addiction. Though glucose regulated protein 78 (GRP78) is a glucose-sensing protein and frequently highly expressed in tumor cells, its roles in glucose and glutamine metabolic regulation remain poorly unknown. We report here that glucose deficiency-induced GRP78 enhances β-catenin signaling and consequently promotes its downstream c-Myc-mediated glutamine metabolism in colorectal cancer cells. Mechanistically, GRP78 elevates intracellular free β-catenin level via disruption of adenomatous polyposis coli (APC)-β-catenin and E-cadherin-β-catenin protein complexes. Notably, overexpression of GRP78 causes APC protein downregulation in proteasome- and lysosome-independent manners. Further mechanistic studies reveal that GRP78 facilitates the extracellular release of APC, thereby rendering the liberation of β-catenin from APC. Furthermore, GRP78 acts through both hindering E-cadherin expression and impairing the interaction of E-cadherin with β-catenin to indirectly and directly influence E-cadherin-β-catenin complex stability. Our study reveals that GRP78 is a novel molecular link between metabolic alterations and signal transduction during tumor progression.

## INTRODUCTION

Compared with normal differentiated cells, cancer cells display ten typical cancer hallmarks, among which an emerging hallmark is the metabolic reprogramming [1]. Cancer cells take up far more glucose than normal cells and switch to glycolysis metabolism even in the presence of ample oxygen, a phenomenon well known as the Warburg effect [2]. Generally, glucose metabolism via aerobic glycolysis provides biosynthetic precursors of nucleic acids, lipids, and proteins as well as reducing power NADPH in rapidly proliferating tumor cells [3-5]. Some cancer cells are addicted to glutamine in support of their survival and growth [6, 7]. Glutamine metabolism via glutaminolysis fuels mitochondrial ATP generation, supplies anapleurotic carbon to the tricarboxylic acid (TCA) cycle, and contributes to the production of NADPH, glutathione (GSH) and lipids [8]. In addition, glutamine also serves as the nitrogen donor for the biosynthesis of nucleotides, hexosamines, and nonessential amino acids [9].

Tumor cell metabolism is sophisticatedly regulated by signal transduction pathways that are affected by genetic mutations or the alterations in tumor microenvironment [10]. PI3K/Akt, HIF-1, c-Myc, p53, and AMPK pathways are intertwined with cancer cell glucose metabolism via affecting the activities or expression of metabolic enzymes and metabolites transporters [10-12], while Rho GTPase/NF-κB, c-Myc and miR-23a/b are implicated in regulating glutamine metabolism involving glutaminase and glutamine transporters [13, 14]. Stimulation of glutamine-dependent mitochondrial anapleurosis results in reduced glucose carbon entering the TCA cycle [14], but promotes glucose uptake and aerobic glycolysis [15]. The presence of glutamine is likely a necessary prerequisite for cancer cells to acquire the aerobic glycolysis phenotype. When glucose metabolism is impaired in glioblastoma cells, the activity of glutamate dehydrogenase is increased to switch glutamine to the TCA cycle and survive impairment of glycolysis [16]. Thus in a reciprocal manner, glutamine metabolism in tumor cells may influence glucose metabolism, and vice versa. However, the mutual regulation mode between glucose metabolism and glutamine metabolism remains to be elucidated in depth.

Glucose regulated protein 78 (GRP78), a molecular chaperone in the endoplasmic reticulum (ER), is also found to be present in tumor cell plasma membrane, cytoplasm, mitochondria, nucleus as well as cellular secretions [17, 18]. GRP78 protein is usually highly induced in poorly perfused solid tumors by the microenvironment factors including hypoxia, acidosis as well as glucose deprivation. High levels of GRP78 contribute to the acquisition of phenotypic cancer hallmarks including apoptosis resistance, immune escape, metastasis and angiogenesis, etc [19]. As a centrally located sensor of microenvironmental stress, an important question, as yet unaddressed, is whether GRP78 is involved in tumor cell metabolism regulation.

In the present study, we identified GRP78 as a molecular link between glucose metabolism and glutamine metabolism. Upon glucose deprivation, the increased levels of GRP78 lead to elevated c-Myc expression and concomitantly to enhanced glutamine metabolism and cell survival from glucose deprivation. These effects of GRP78 are attributed to the elevated β-catenin protein levels, mediated by interference with the adenomatous polyposis coli (APC)-β-catenin and E-cadherin-β-catenin complexes. Specifically, overexpression of GRP78 impairs the expression of E-cadherin and its interaction with β-catenin to influence E-cadherin-β-catenin complex stability. Interestingly, GRP78 is able to facilitate the extracellular release of APC via protein-protein interaction, consequently resulting in the liberation of β-catenin from APC. Our results thus reveal a novel complementary pathway between glucose metabolism and glutamine metabolism. It may hold important implications for strategies aiming at targeting tumor metabolism.

## RESULTS

### c-Myc-mediated glutamine metabolism promotes cell survival from glucose deprivation

**Figure 1:**
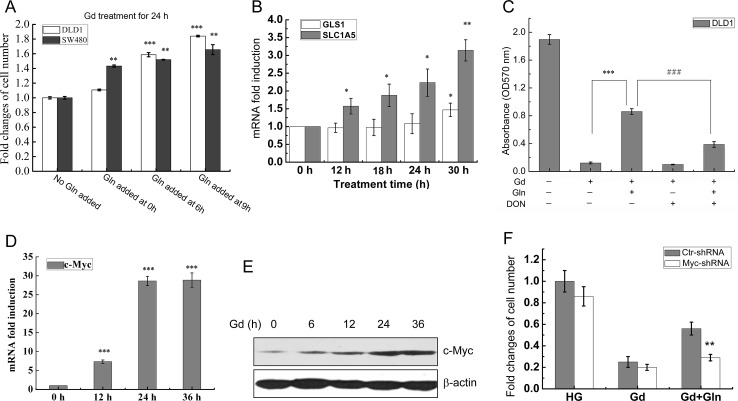
Myc-mediated Glutamine metabolism promotes cell survival from glucose deprivation (A) DLD1 and SW480 cells were exposed to glucose deprivation (0.5 mM), and glutamine was added after Gd treatment for 0, 6 and 9 h, respectively. Cell viabilities were evaluated by MTT assay (mean±SEM). **p<0.01, ***p<0.001 vs Gd (n=3). (B) Relative mRNA levels of GLS1 and SLC1A5 in DLD1 cells at the indicated time points following Gd treatment. *p<0.05, **p<0.01 vs 0 h. (C) DLD1 cells were exposed to Gd in the presence of glutamine and DON. The cell number was represented by the absorbance at 570 nm. ***p<0.001 vs Gd. ###p<0.001 vs Gd+Gln (n=3). (D) Relative mRNA levels of c-Myc in DLD1 cells at the indicated time points following Gd treatment. ***p<0.001 vs 0 h. (E) Representive Western blots of c-Myc in DLD1 cells treated with Gd for different time intervals. (F) DLD1 cells were transfected with either Ctrl-shRNA and Myc-shRNA and subjected to growth under Gd conditions with or without glutamine for 24 h. The cell number changes were examined by MTT assay. **p<0.01 vs Ctr-shRNA.

Previous studies demonstrated that glioblastoma cells required the glutamate dehydrogenase (GDH) to drive glutamine metabolism to the TCA cycle and survived glucose metabolism impairment [16]. To determine the role of glutamine in colorectal cancer cell survival upon glucose deprivation, 10 mM glutamine was added at 0, 6 and 9 hours after glucose deprivation (Gd). MTT assay, microscopy observation and flow cytometry indicated that glutamine addition significantly promoted cell survival in DLD1 and SW480 cells (Fig. 1A, S1A and S1B). Surprisingly, addition of glutamine to DLD1 cells grown in high glucose medium retarded the cell growth (Fig. S1D), suggesting that the effects of glutamine on cell growth is affected by the glucose supply.

We next observed that the mRNA levels of both glutaminase 1 (GLS1) and glutamine transporter SLC1A5 (ASCT2) but not GDH were increased in response to glucose deprivation (Fig. 1B and S1E). Treatment with 6-diazo-5-oxo-L-norleucine (DON), an inhibitor of glutaminase, largely reversed the prosurvival effect of glutamine (Fig. 1C). c-Myc has been reported to directly regulate SLC1A5 mRNA transcription and indirectly regulate GLS1 through suppression of miR-23 [14, 20]. Consistently with the above data, both c-Myc mRNA and protein levels were considerably elevated in Gd-treated DLD1 cells (Fig. 1D and 1E), while knockdown of c-Myc by lentiviral shRNA partially reversed the prosurvival effect of glutamine (Fig. 1F and S1C). These data indicate that glutaminase-dependent glutamine metabolism can retard Gd-induced cell death, and this effect is at least partly mediated by Gd-stimulated c-Myc expression.

### Glucose deprivation-induced c-Myc expression is mediated by β-catenin

**Figure 2:**
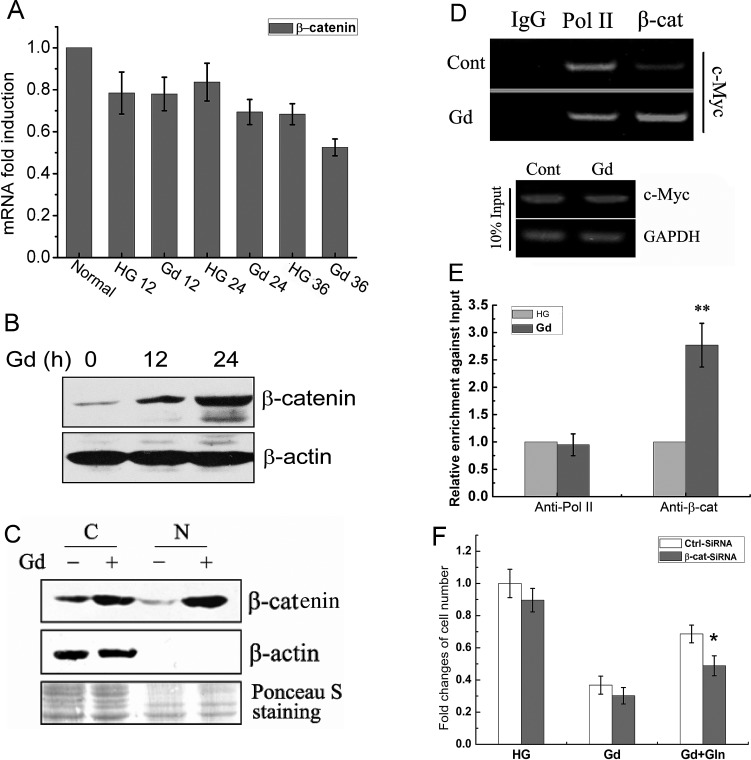
Glucose deprivation-induced c-Myc expression is mediated by β-catenin (A) Relative mRNA levels of β-catenin in DLD1 cells at the indicated time points following Gd treatment (n=3). (B) Representive Western blots of β-catenin in DLD1 cells exposed to Gd for 12 h and 24 h. (C) Western blotting analysis of β-catenin levels in cytosolic and nuclear extracts of the cells treated with/without Gd. (D) CHIP of the c-Myc promoter using antibody against β-catenin in DLD1 cells. Control mouse IgG and antibody against Pol II were used as the negative control and positive control, respectively. Inputs were PCR products from DNA extracts without CHIP. (E) Quantitative enrichment of and β-catenin at c-Myc promoter versus the input samples. **p<0.01 vs anti-Pol II. (F) DLD1 cells were transfected with either Ctrl-siRNA and β-catenin-siRNA and subjected to growth under Gd conditions in the presense or absence of glutamine for 24 h. The cell number changes were examined by MTT assay. *p<0.05 vs Ctrl-siRNA.

Given that c-Myc is a direct target gene of the β-catenin pathway [21], the expression of β-catenin mRNA and protein levels were examined in Gd-treated DLD1 cells. As shown in Fig. 2A and 2B, there was a slight decrease in β-catenin mRNA levels but a marked increase in its protein level after 12 h and 24 h of Gd treatment, suggesting that the regulation of β-catenin by Gd stimulation occurs at post-transcriptional level. Western blotting analysis of cytoplasmic and nuclear extracts further revealed the accumulation of β-catenin in both nuclear and cytosolic fractions of Gd-stimulated DLD1 cells (Fig. 2C). Moreover, the increased β-catenin nuclear localization was accompanied by enhanced binding between β-catenin and c-Myc promoter sequence as revealed by ChIP assay (Fig. 2D and 2E). Knockdown of β-catenin by siRNA in DLD1 cells reduced Gd-induced c-Myc expression (Fig. S2A) and partially reversed the prosurvival effect of glutamine under Gd conditions (Fig. 2F), which mimics the effects of c-Myc knockdown. Taken together, these data demonstrate that glucose deprivation can elevate the protein level of β-catenin, which further transactivates its downstream target gene c-Myc.

### Glucose deprivation-induced GRP78 elevates β-catenin protein level

**Figure 3:**
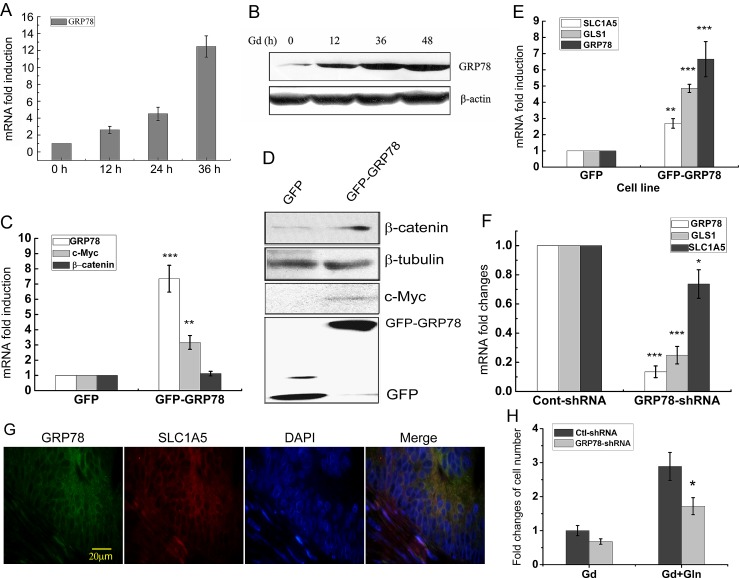
Glucose deprivation-induced GRP78 elevates β-catenin protein level (A) Relative mRNA levels of GRP78 in DLD1 cells at the indicated time points following Gd treatment. (B) Western blots of GRP78 in DLD1 cells exposed to Gd for 0, 12, 24 and 36 h. (C) Relative mRNA levels of GRP78, c-Myc and β-catenin in GRP78 overexpressing DLD1 cells. **p<0.01, ***p<0.001 vs GFP. (D) Western blots of c-Myc, β-catenin and GFP in GRP78 overexpressing DLD1 cells. (E) Relative mRNA levels of GRP78, GLS1 and SLC1A5 in GRP78 overexpressing DLD1 cells. **p<0.01, ***p<0.001 vs GFP. (F) Relative mRNA levels of GRP78, GLS1 and SLC1A5 in GRP78 knockdown DLD1 cells. *p<0.05, ***p<0.001 vs Cont-shRNA. (G) Fluorescent immunohistochemical demonstration of GRP78 and SLC1A5 in a 5-μm paraffin-embedded section. (H) DLD1 cells were transfected with either Ctrl-shRNA and GRP78-shRNA and subjected to growth under Gd conditions with or without glutamine for 24 h. The cell number changes were examined by MTT assay. *p<0.05 vs Ctrl-shRNA.

**Figure 4:**
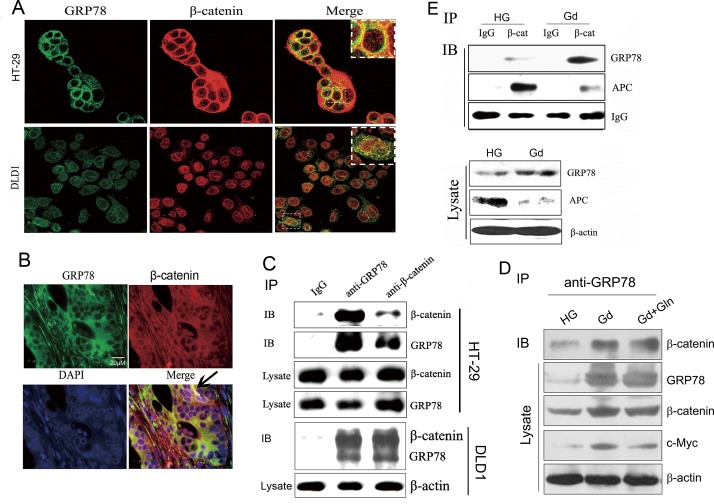
GRP78 co-localizes with β-catenin in colorectal cancer cells (A) HT-29 and DLD1 cells were stained with antibodies against GRP78 and β-catenin, and visualized with FITC- and TRITC-conjugated secondary antibodies, respectively. The yellow dots correspond to spots where GRP78 (green) and β-catenin (red) colocalized. (B) Fluorescent immunohistochemical demonstration of GRP78 and β-catenin in a 5-μm paraffin-embedded section. (C) Analysis of GRP78 and β-catenin interactions. Immunoprecipitation experiments were carried out on HT-29 (top) and DLD1 (bottom) lysates using GRP78 and β-catenin antibodies followed by Western blotting for β-catenin and GRP78. (D) Analysis of GRP78 and β-catenin interactions under the conditions of Gd treatment with or without glutamine addition. Immunoprecipitation experiments were carried out on DLD1 lysates using anti-GRP78 antibodies followed by Western blotting detection of β-catenin. (E) Co-immunoprecipitation assay was performed on DLD1-derived cell lysates after growth in HG or Gd condition for 24 h, using anti-β-catenin antibody and followed by Western blotting for GRP78 and APC.

GRP78 protein is an important glucose-regulated protein and contributes to the acquisition of several cancer hallmarks by tumor cells [19, 22]. Both GRP78 mRNA and protein levels were persistently upregulated throughout the time course of Gd treatment (Fig. 3A and 3B). Furthermore, addition of glutamine did not significantly affect the expression of GRP78 and c-Myc induced by Gd treatment (Fig. 4D and S2B). To investigate whether GRP78 is implicated in regulating glutamine metabolism under Gd conditions, GRP78 was overexpressed or knocked down in DLD1 cells by lentivirus gene transfer. GRP78 overexpression (GFP-GRP78) significantly raised β-catenin protein level, concurrently accompanied by elevated c-Myc mRNA and protein levels (Fig. 3C and 3D). GRP78 overexpression also caused the increased mRNA expression of GLS1 and SLC1A5 (Fig. 3E), while knockdown of endogenous GRP78 expression in DLD1 cells by shRNA (GRP78-shRNA) significantly reduced the mRNA levels of GLS1 and SLC1A5 (Fig. 3F). Consistent with the above results, GRP78 and SLC1A5 are positively correlated in human colon cancer xenograft tissue in SCID mice [23] (Fig. 3G). Importantly, GRP78 knockdown could partially reverse the prosurvival effects of glutamine under Gd conditions (Fig. 3H). These results indicate that Gd-induced glutamine metabolic alterations can be phenocopied by GRP78 overexpression, suggesting that the prosurvival effect of glutamine metabolism under Gd conditions is at least partially mediated by Gd-induced GRP78 expression.

### GRP78 co-localizes with β-catenin in colorectal cancer cells

In epithelial cells, β-catenin is mainly localized in the cytoplasm, complexed with APC and Axin, and in cell-cell junction in conjunction with E-cadherin [24]. How would GRP78 function in regulation of β-catenin protein level? One possibility is that GRP78 may promote β-catenin stability via protein-protein interaction. Confocal immunofluorescence results implicated that GRP78 co-localized with β-catenin in HT-29 and DLD1 cells (Fig. 4A) as well as in human colon cancer xenograft tissue (Fig. 4B). Co-immunoprecipitiation (co-IP) assays with antibody against GRP78 revealed that β-catenin complexed with GRP78 in HT-29 and DLD1 cells. Similarly, the reverse precipitation using a β-catenin antibody also showed that GRP78 complexed with β-catenin (Fig. 4C). Co-IP and immunocytochemical staining assays further showed that protein complexes between GRP78 and β-catenin were increased, while the interaction between β-catenin and APC was decreased in response to Gd treatment (Fig. 4E and S3). In addition, the enhanced interaction between GRP78 and β-catenin stimulated by Gd treatment was not appreciably affected by glutamine addition (Fig. 4D). Interestingly, the amount of APC protein in the whole cell lysate was reduced after Gd treatment, which is likely to account for the decreased interaction between β-catenin and APC (Fig. 4E).

### Gd-induced APC downregulation is mediated by GRP78 and is independent of proteasomal and lysosomal systems

**Figure 5:**
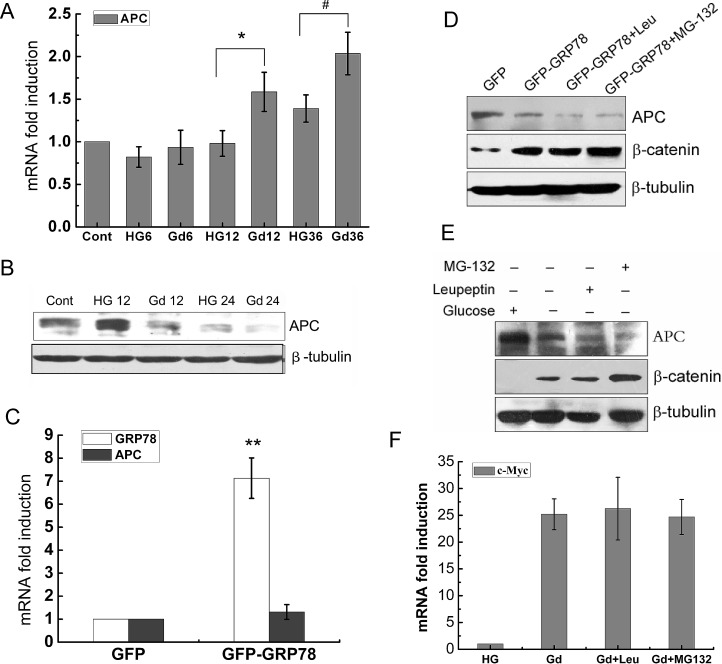
Gd-induced APC downregulation is mediated by GRP78 and is independent of proteasomal and lysosomal systems (A) Relative mRNA levels of APC in DLD1 cells at the indicated time points following Gd treatment. *p<0.05 vs HG12. #p<0.05 vs HG36. (B) Western botting analysis of APC expression in DLD1 cells after growth in high glucose or glucose deprivation for 12 and 24 h. (C) Relative mRNA levels of APC in GRP78 overexpressing DLD1 cells. **p<0.01 vs GFP. (D) Western botting analysis of APC and β-catenin expression in GRP78 overexpressing DLD1 cells in the presence and absence of MG-132 or leupeptin. (E) Western botting analysis of APC and β-catenin expression in Gd-stimulated DLD1 cells in the presence and absence of MG-132 or leupeptin. (F) Relative mRNA levels of c-Myc in Gd-stimulated DLD1 cells in the presence and absence of MG-132 or leupeptin.

The downregulation of APC protein in Gd-treated cells led us to test whether it occurred at transcriptional level or posttranscriptional level. The APC mRNA levels in Gd-treated cells were moderately increased in comparison to cells grown in high glucose medium, while its protein levels were decreased at the corresponding treatment time points (Fig. 5A and 5B). Therefore, Gd-induced APC protein reduction is not due to a transcriptional regulation. GRP78 overexpression (GFP-GRP78) also caused a slightly increase of APC mRNA level but induced a decline of APC protein level, which phenocopied the effects of Gd treatment (Fig. 5C and 5D). These data suggest that Gd-induced APC downregulation is mediated by GRP78 expression.

The proteasomal and lysosomal pathways are the two main routes of protein and organelle clearance in eukaryotic cells [25]. To further elucidate the mechanism underlying the downregulation of APC protein under Gd conditions, proteasome inhibitor MG-132 and lysosomal proteases inhibitor leupeptin were used to inhibit these two pathways. As shown in Fig. 5D and 5E, neither MG-132 nor leupeptin could abrogate the regulatory effects of Gd treatment or GRP78 overexpression on the APC protein. In addition, Gd-induced c-Myc expression was not affected by MG-132 and leupeptin treatment either (Fig. 5F). Together, these data indicate that the downregulation of APC protein by Gd treatment or GRP78 overexpression is independent of proteasomal and lysosomal protein degradation systems.

### GRP78 induces APC downregulation via promotion of APC secretion

**Figure 6:**
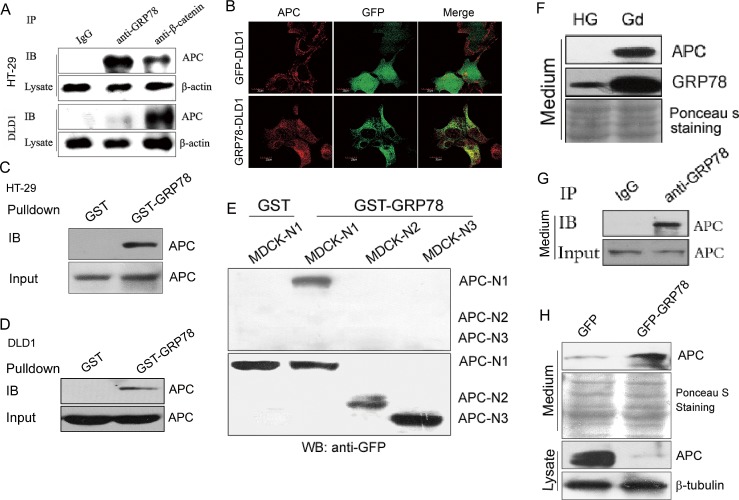
GRP78 induces APC downregulation via promotion of APC secretion (A) Analysis of GRP78 and APC interactions. Immunoprecipitation experiments were carried out on HT-29 (top) and DLD1 (bottom) lysates using anti-GRP78 and anti-β-catenin antibodies followed by Western blotting for APC. (B) APC was stained in DLD1 cells stably expressing GFP and GFP-GRP78 fusion protein. (C) Cell lysates from HT-29 cells were incubated with GST or GST-GRP78 glutathione beads. The precipitates were separated by SDS-PAGE and immunoblotted with anti-APC antibody. (D) GST-GRP78 pulldown of lysates from DLD1 cells. (E) GST-GRP78 pulldown of lysates from MDCK cells stably expressing the truncated N1-APC (aa 1-448), N2-APC (aa 449-781) and N3-APC (782-1082) respectively. The precipitates were separated by SDS-PAGE and immunoblotted with anti-GFP antibody. (F) Western botting analysis of APC and GRP78 secretion in conditioned medium from DLD1 cells grown with/without glucose. (G) Analysis of GRP78 and APC interactions in conditioned medium. Immunoprecipitation experiments were performed on glucose free conditioned medium from DLD1 cells using GRP78 antibody followed by Western blotting for APC. (H) Western botting analysis of APC in conditioned medium from DLD1 cells stably expressing GFP and GFP-GRP78 fusion protein.

Given that GRP78 has been reported to be secreted from solid tumor cells into the tumor microenvironment [18], we speculated that GRP78 secretion may lead to the extracellular release and cytosolic downregulation of APC protein. To test this possibility, Co-IP assays were first performed to detect the interaction between GRP78 and APC. As shown in Fig. 6A, GRP78 could co-immunoprecipitate with APC in both HT-29 and DLD1 cells. Immunocytochemical staining also showed the co-localization of APC with GFP-GRP78 protein but not with GFP in transfected DLD1 cells (Fig. 6B). GST-pulldown assays demonstrated that purified recombinant GST-GRP78 protein bound to the APC protein from both DLD1 and HT-29 cell extracts (Fig. 6C and 6D). To further determine the interacting domain of APC with GRP78, GST pulldown assays were performed with lysates of MDCK cells stably expressing the truncated N1-APC (aa 1-448), N2-APC (aa 449-781) and N3-APC (782-1082) respectively. The results showed that GST-GRP78 protein only bound to the truncated N1-APC but not the N2-APC and N3-APC (Fig. 6E). After showing the interaction between GRP78 and APC protein, we intended to investigate whether the APC protein could be secreted from colon cancer cells. As shown in Fig. 6F, Gd treatment increased the secretion of GRP78 protein, which was accompanied by the obvious extracellular secretion of APC. Furthermore, GRP78 co-immunoprecipitated with APC in the medium from DLD1 cells (Fig. 6G). Consistently, GRP78 overexpression (GFP-GRP78) also resulted in increased extracellular secretion of APC, which phenocopied the effects of Gd treatment (Fig. 6H). We have identified that sodium butyrate (SB) treatment can block the membrane translocation and extracellular secretion of GRP78 (data not shown). Exposure to SB resulted in a decrease of APC in GFP-DLD1 cells but not in GRP78-DLD1 cells (Fig. S4A), suggesting that impaired GRP78 secretion results in cytoplasmic accumulation of APC. We further showed that both GRP78 and APC colocalized with CD63 (Fig. S4B and S4C), a marker of late multivesicular endosomes (MVEs) or secreted exosome vesicles [26], while treatment with SB resulted in the aggregation of GRP78 protein in cells and markedly diminished colocalization of GRP78 and APC with CD63 (Fig. S4D). The results above suggest that Gd treatment promotes the expression and secretion of GRP78, which further leads to the extracelluar secretion of APC by means of protein-protein interaction.

### GRP78 overexpression interferes with the E-cadherin-β-catenin complex

**Figure 7:**
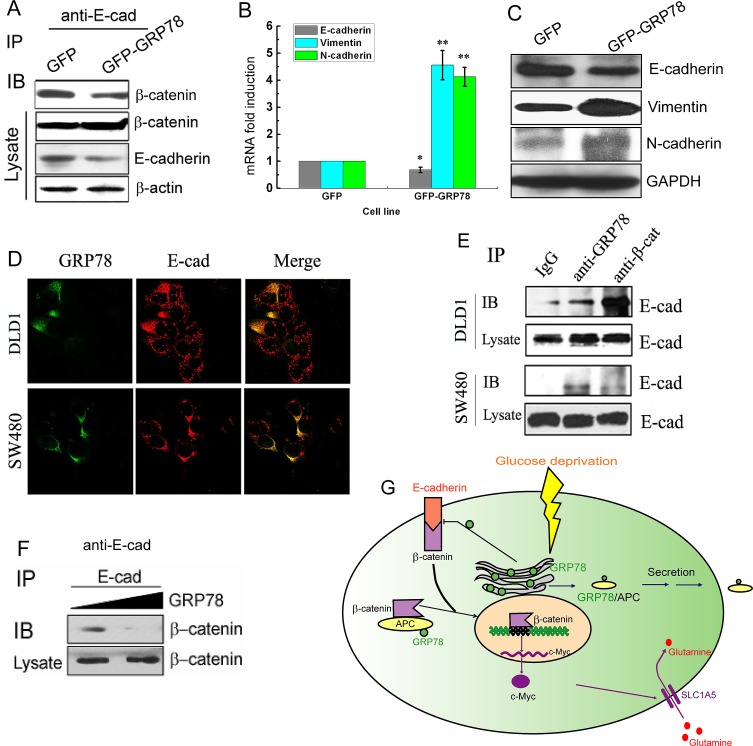
GRP78 overexpression interferes with the E-cadherin-β-catenin complex (A) Analysis of E-cadherin and β-catenin interactions in GRP78 stably expressing DLD1 cells by Co-IP assay. Immunoprecipitations were performed using anti-E-cadherin antibody and followed by Western blotting for β-catenin. (B) Relative mRNA levels of E-cadherin, N-cadherin and vimentin in GRP78 stably expressing DLD1 cells. (C) Western botting analysis of E-cadherin, N-cadherin and vimentin expression in GRP78 overexpressing DLD1 cells. (D) Double staining of GRP78 and E-cadherin in DLD1 and SW480 cells with antibodies against GRP78 and E-cadherin, followed by visualization with FITC and TRITC, respectively. (E) Analysis of E-cadherin and GRP78 interactions in DLD1 and SW480 cells by Co-IP assay. Immunoprecipitations were performed using anti-GRP78 and anti-β-catenin antibodies and followed by Western blotting for E-cadherin. (F) Ineracting changes of E-cadherin and β-catenin after exogenous addition of GRP78 protein. Immunoprecipitation experiments were performed on DLD1 cell lysates after adding different amounts of purified GRP78 protein, using anti-E-cadherin antibody, followed by Western blotting for β-catenin. (G) Schematic representation of the proposed model. Solid tumors frequently outgrow the blood supply, resulting in nutrient insufficiency, such as glucose deficiency, which induces GRP78 expression in cancer cells. One one hand, GRP78 directly interacts with APC in the cytoplasm, and thus APC can be sorted into the exosome and released into the extracellular space along with the GRP78 protein, thereby relieving the negative regulation of APC on β-catenin. One the other hand, GRP78 acts through both hindering E-cadherin expression and impairing the interaction of E-cadherin with β-catenin to influence E-cadherin-β-catenin complex stability. The increased free β-catenin in the cytoplasm will lead to activation of its target gene c-Myc, which stimulates the glutamine metabolim and promote cell survival from glucose impairment.

Besides in the β-catenin-destruction complex, β-catenin is also localized at the adherens junction complexing with E-cadherin and α-catenin [27]. We next dedicated to investigate whether GRP78 could affect the complex formation of β-catenin with E-cadherin. As shown in Fig. 7A, the interaction between E-cadherin and β-catenin protein became weak in GRP78 stably expressing cells. GRP78 overexpression (GFP-GRP78) lead to the decreased E-cadherin expression at both mRNA and protein levels but the increased N-cadherin and vimentin expression (Fig. 7B and 7C), suggesting that GRP78 overexpression can indirectly disrupt the E-cadherin-β-catenin complex through inhibition of E-cadherin expression.

We next asked whether GRP78 could directly interfere with the β-catenin-E-cadherin complex. Immunocytochemical staining showed an obvious colocalization of GRP78 with E-cadherin (Fig. 7D). Co-IP assays also showed that GRP78 could co-immunoprecipitate with E-cadherin in both SW480 and DLD1 cells (Fig. 7E). Moreover, when the purified GRP78 protein was added to the cellular lysate, the interaction between E-cadherin and β-catenin became weak (Fig. 7F). Taken together, these data demonstrate that GRP78 can obstruct E-cadherin-β-catenin complex in both indirect and direct manners, both of which can theoretically result in increased free β-catenin levels.

## DISCUSSION

Glucose and glutamine are abundant in plasma, and supply the carbon, nitrogen, energy, and reducing equivalents required for tumor cell growth and division [28]. Targeting tumor metabolism may be a promising strategy to develop new types of anti-cancer drugs. However, the premise is that only by thoroughly dissecting the processes of metabolic modulation can we explore them as targets for cancer therapy. Evidence has been presented that glutamine metabolism via mitochondrial TCA cycle play a complementary effect on the survival of B cells and glioblastoma cells under glucose deprivation [16, 29]. In this study, we present a more direct molecular link between glucose metabolism and glutamine metabolism in colorectal cancer cells: glucose deficiency induces GRP78 expression, which increases free β-catenin protein level through impairing the β-catenin-E-cadherin and β-catenin-APC complexes. The elevated free β-catenin results in increased c-Myc expression, with consequent augment of glutamine metabolism.

Deregulation of Wnt/β-catenin pathway usually occurs in colorectal cancer, which leads to a constitutively stable and active β-catenin and induces aberrant cell proliferation [30]. Under hypoxia conditions, β-catenin binds to hypoxia inducible factor-1α (HIF-1α) and enhances HIF-1-mediated transcription, thereby promoting cell survival and adaptation to hypoxia [31]. Our present data indicate that β-catenin also functions as a prosurvival signal via promotion of c-Myc-mediated glutamine metabolism in the face of glucose deficiency. Thus the complex β-catenin signal activities not only fuel cell growth and proliferation but also support cell survival under the unfavourable conditions encountered in the tumor microenvironment.

There is solid evidence that GRP78 plays a role in resisting stressful microenvironment and facilitating cell survival, and the underlying mechanism involves blockage of pro-apoptotic pathways or activation of pro-survival pathways [32]. Our data suggest that under glucose limited conditions, the induced GRP78 can motivate glutamine metabolism to compensate for the impairment of glucose metabolism and prolong cell survival. The survival-promoting effect of GRP78 via metabolism modulation is independent of the classical apoptotic or survival signaling pathways.

GRP78 is multifunctional protein, and what makes GRP78 more special is that GRP78 can be present on cancer cell surface or secreted into extracellular microenvironment. The present data demonstrate that these features are intertwined with the modulation of β-catenin-E-cadherin and β-catenin-APC protein complexes. High expression of GRP78 can reduce E-cadherin expression through induction of epithelial-mesenchymal transition (Fig. 7B and 7C). Futhermore, membrane translocation of GRP78 appears to directly disrupt the β-catenin-E-cadherin complex through an as yet unidentified mechanism.

Full-length APC is a β-catenin binding protein and can down-regulate β-catenin-mediated transcription. However, different APC truncations freguently happen in colorectal cancer [33], which usually lead to different molecular consequences on β-catenin regulation [34]. Our results reveal an unexpected finding that glucose deficiency is able to stimulate the extracellular secretion of APC, causing the reduction of its intracellular levels. This effect is mediated by GRP78 via protein-protein interaction. GRP78 interacts with APC in both the intracellular and extracellular medium, and that both GRP78 and APC colocalize with the exosome marker CD63. Though the detailed secretion process of APC is not yet unknown, the most likely scenario seems to be that GRP78 protein and its tethered APC protein are jointly sorted into the multivesicular endosomes and subsequently released into the extracellular microenvironment in the form of exosome-like vesicles.

APC is a large protein, with multi-functional domains, implicated in a wide range of biological processes. The COOH-terminal domain of APC (C-APC) interacts with its NH2-terminal domain (N-APC), and this interaction can cause changes in the protein interactions of N-APC [35]. Our data demonstrate that GRP78 interacts with APC at its N-terminus in colon cancer cells whose COOH-terminal domains of APC are missing. Thus whether the secretory regulation of APC by GRP78 is affected by C-APC and only happens in cells with C-terminal truncations is a question remains to be confirmed in further investigations. In addition, secreted GRP78 in tumor microenvironment is likely to be involved in immune regulation and drug resistence [18, 19]. Whether the cellular released APC in tumor milieu can function in cell communication is another fascinating topic to be further addressed.

## MATERIALS AND METHODS

### Materials

RPMI-1640 medium and fetal bovine serum (FBS) were from GIBCO (Grand Island, NY). Trizol, PrimeScript RT Master Mix and SYBR green PCR master mix were from Takara (Shiga, Japan). Antibodies for E-cadherin and β-actin were from Bioworld Technology (Minneapolis, MN). Antibodies for β-tubulin, GFP, GRP78 and APC were from Abcam (Cambridge, UK). Antibodies for β-catenin and CD63 were obtained from Abmart (Shanghai, China) and Santa Cruz Biotechnology (Santa Cruz, CA), respectively. FITC-, TRITC-, Cy5-, and HRP-conjugated secondary antibodies were obtained from Invitrogen (Carlsbad, CA).

### Cell culture and cell number determination

Human colon carcinoma HT29, SW480 and DLD1 cell lines were obtained from the American Type Culture Collection and cultured in RPMI-1640 medium containing 10% FBS at 37 °C in humidified tissue culture incubator containing 5% CO_2_. MDCK cells stably expressing the truncated N1-APC (aa 1-448), N2-APC (aa 449-781) and N3-APC (782-1082) were the gift from Prof. Inke Näthke (University of Dundee, UK). The viability of cells was assessed by the MTT assay as the literature described [36].

### RNA extraction and real-time PCR analysis

Total RNA extraction, reverse transcription, and real-time PCR were performed as previously described [37]. The primers used in this study were depicted in supplementary Table 1.

### Nuclear Protein Extraction and Western blotting

The nuclear and cytosolic proteins were extracted using a nuclear protein extraction kit (NE-PER™, Pierce). The protein concentrations of nuclear, cytosolic and whole cell lysates were determined by the BCA protein assay. An appropriate amount of protein from each sample was subjected to SDS-PAGE and subsequent Western blotting as previously described [37].

### Co-immunoprecipitation and GST-pulldown assays

Cells were lysed in Western and IP buffer (Beyotime, China) containing protease inhibitor cocktail (Thermo scientific). The whole cell lysates (WCL) were pre-cleared by incubating with 1.0 μg of control IgG corresponding to the host species of primary antibody, together with 20 μl Protein A/G PLUS-Agarose (Santa Cruz) at 4 °C for 2 h, followed by centrifugation at 4°C for 5 min at 2, 500 rpm. The supernatant was subjected to immunoprecipitation by addition of 2 μg immunoprecipitation antibodies and incubated overnight at 4 °C followed by incubation with Protein A/G PLUS-Agarose for 2 h. After washing with cell lysis buffer, the beads were boiled in 2×SDS loading buffer and the supernatants were resolved by SDS-PAGE. For the GST-pulldown assays, GST-GRP78 or GST immobilized on Sepharose 4B-glutathione beads (GE Healthcare) were incubated with pre-cleared WCL at 4 °C for 12 h followed by washing with the lysis buffer. The bound proteins were dissolved in 2×SDS loading buffer, separated by 10% SDS-PAGE, and immunoblotted with the APC antibody.

### Immunofluorescence analysis

Cells were plated on 6-well glass slides. After the required treatments, the cells were then fixed in 4% paraformaldehyde in PBS for 30 min, and permeabilized with 0.3% Triton X-100 in PBS for 10 min. Next, the slides were blocked in 2% goat serum for 1 h and incubated with the primary antibodies at 4 °C overnight. The slides were then washed and incubated with the corresponding secondary antibodies. After three PBS washes, the slides were mounted in gelvatol for confocal immunofluorescence analysis.

For tissue samples, paraffin-embedded sections were deparaffinized in xylene and rehydrated through descending concentrations of ethanol. After routine antigen retrieval procedures, sections were treated with 10% FBS for 30 min to block the nonspecific binding. Sections were then incubated with the appropriate diluted primary antibodies and secondary antibodies as described above. The sections were finally counter stained with DAPI and mounted in gelvatol for immunofluorescence analysis.

### Chromatin immunoprecipitation (CHIP)

CHIP assay was performed using a chromatin immunoprecipitation kit (EZ-CHIPTM, Upstate) following the manufacturer's instruction. Briefly, 1×10^7^ formaldehyde crosslinked DLD1 cells were sonicated for DNA shearing to achieve DNA fragments of about 200-1000 bp. Immunoprecipitation was carried out using anti-β-catenin antibody, anti-pol II antibody (positive control) and the control IgG (negative control) overnight at 4°C. The primers used to amplify the DNA fragments were depicted in Supplementary Table 2.

### Lentivirus infection

Human GRP78 cDNA was subcloned into the lentiviral expressing vector pLVX-AcGFP1-N1 Vector (Clontech). GRP78 shRNAs constructed in pLKO.1-Puro were purchased from Sigma (Mission shRNA). GRP78 overexpression/shRNA plasmid, pCMVdR8.91 and pCMV-VSV-G were co-transfected into 293T cells using the Calcium Phosphate method at 15:10:5 g (for a 10-cm dish). Media containing virus was collected 48 h after transfection and then concentrated using 100 kDa ultrafiltration membranes (Millipore). DLD1 cells were infected with the viruses in the presence of polybrene (8 μg/ml) for 48 h, and then subjected to selection by 5 μg/ml puromycin for 72 hours. Hairpin sequences in these shRNA constructs are depicted in Supplementary Table 3.

### Statistical analysis

Data are expressed as the mean ± SEM. Differences among groups were tested by one-way analysis of variance (ANOVA). Comparisons between two groups were evaluated using Student's t-test. A value of p<0.05 was considered statistically significant.

## SUPPLEMENTARY MATERIAL FIGURES AND TABLES


