# Supplementary material for: GRP78 enhances the glutamine metabolism to support cell survival from glucose deficiency by modulating the β-catenin signaling
Source: Oncotarget. 2014 Jun 14;5(14):5369–80. doi: 10.18632/oncotarget.2105 (PMC4170599; doi:10.18632/oncotarget.2105)
Supplement: Supplementary file 1 [file oncotarget-05-5369-s001.pdf]

GRP78 enhances the glutamine metabolism to support cell survival from glucose deficiency by modulating the β-catenin signaling

Supplementary Material

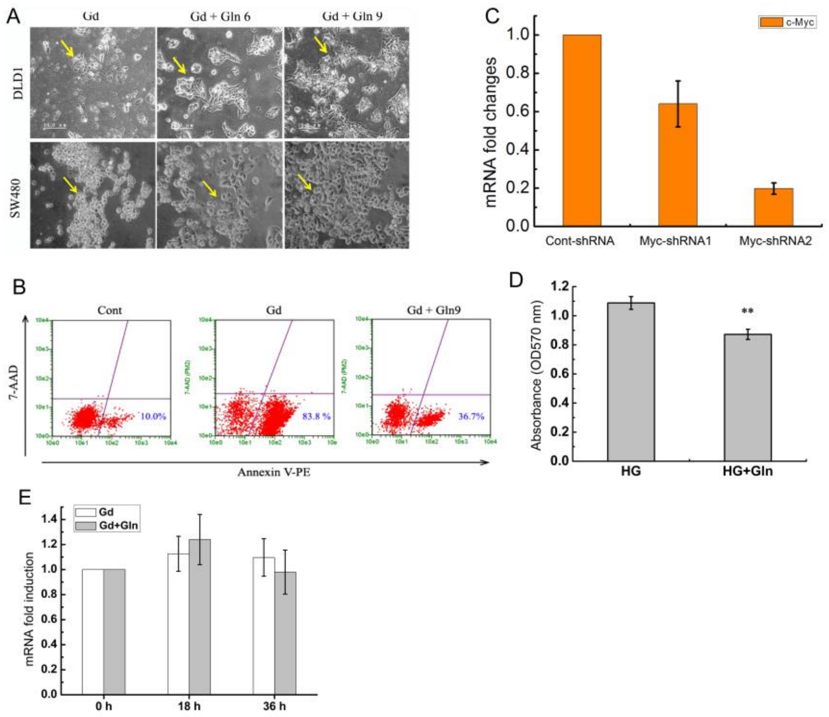

Figure S1: Effects of glutamine on tumor cell growth in glucose-deprived and high glucose conditions. (A) DLD1 and SW480 cells were exposed to glucose deprivation (Gd), and glutamine was added after Gd treatment for 6 and 9 h, respectively. The photographs of morphological changes were taken using phase-contrast inverted microscopy. (B) Flow cytometric analysis of apoptotic cells after Annexin V-PE and 7-AAD staining. Viable cells are Annexin V-PE$^-$/7-AAD$^-$. Annexin V-PE$^+$/7-AAD$^-$ cells are in the early apoptotic phase, whereas the Annexin V-PE$^+$/7-AAD$^+$ cells are in the late apoptotic phase. Necrotic cells are shown as Annexin V-PE$^-$/7-AAD$^+$. (C) Relative mRNA levels of c-Myc in DLD1 cells infected with lentivirus expressing nontargeting shRNA or c-Myc-targeting shRNA. (D) DLD1 cells were grown in high glucose medium in the presence and absence of 10 mM glutamine for 24 h. Cell growth were evaluated by MTT assay (mean±SEM). $^{**}p<0.01$ vs HG. (E) Relative mRNA levels of GDH in DLD1 cells at the indicated time points following Gd treatment in the presence and absence of Gln.

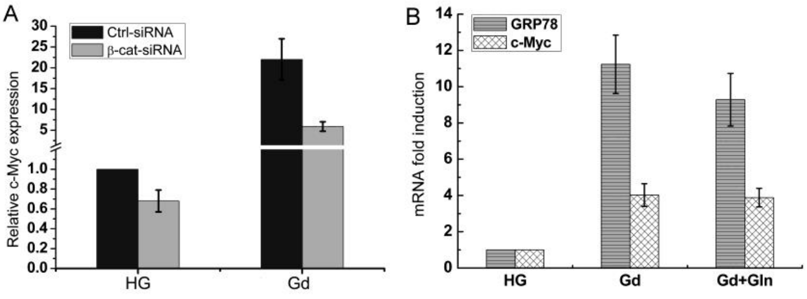

Figure S2: The relative mRNA levels of c-Myc and GRP78 by real-time PCR assay. (A) Relative mRNA changes of c-Myc before and after β-catenin knockdown under the conditions of high glucose (HG) and glucose deprivation (Gd). (B) Relative mRNA levels of GRP78 and c-Myc under the conditions of HG, Gd and Gd with glutamine added (Gd+Gln).

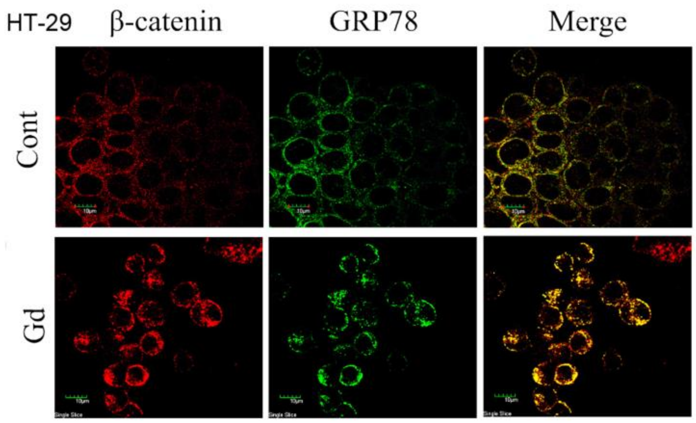

Figure S3: Glucose deprivation enhances the co-localization of GRP78 and β-catenin. (A) HT-29 cells were stained with antibodies against GRP78 and β-catenin, and visualized with FITC- and TRITC-conjugated secondary antibodies, respectively. The yellow dots correspond to spots where GRP78 (green) and β-catenin (red) colocalized.

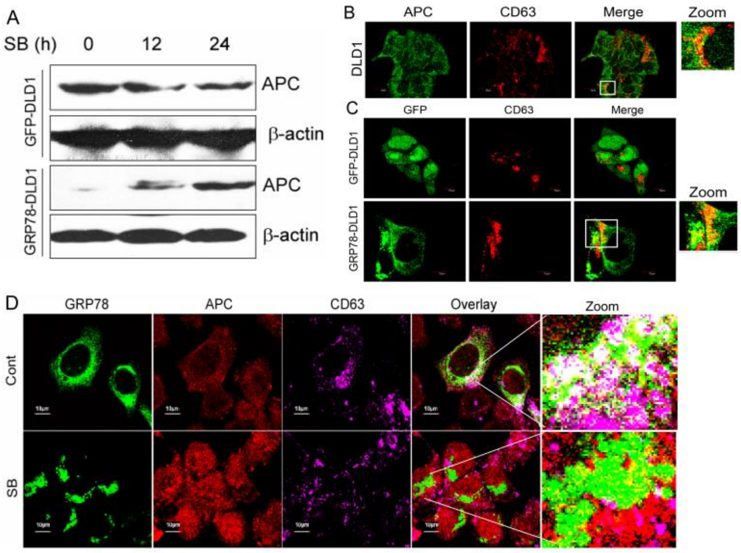

Figure S4: GRP78 and APC colocalize with the exosome marker CD63. (A) Representative Western blots of APC and β-actin in DLD1 cells stably expressing GFP and GFP-GRP78 fusion protein. The cells were treated with 2 mM sodium butyrate (SB) for 0, 12 and 24 h. (B) DLD1 cells were stained with antibodies against APC and CD63, and visualized with FITC- and TRITC-conjugated secondary antibodies, respectively. (C) CD63 was stained and visualized using TRITC-conjugated secondary antibody in DLD1 cells stably expressing GFP and GFP-GRP78 fusion protein. (D) APC and CD63 were stained in DLD1 cells stably expressing GFP or GFP-GRP78, and were visulized with TRITC- and Cy5-conjugated secondary antibodies, respectively.

Table S1: List of primers used in this study

Primers	*Sequence*	
	Fwd	*Rev*
β-catenin	GCTGAAGGTGCTATCTGTCTGCTC	TGAACAAGACGTTGACTTGGATCTG
GLS1	CAGCCTCCAGGTGCTTTCA	GAACATTGGCCGTGGTTTCA
GLC1A5	TCCTGGCTGTGGACTGGCTA	TGTATCAACTCAGGCTCTGTGCTTC
c-Myc	CTGCGACGAGGAGGAGAA	CCGAAGGGAGAAGGGTGT
GRP78	CTGTGCAGCAGGACATCAAGTTC	TGTTTGCCCACCTCCAATATCA
APC	TTACAGTCCCAAGCAACA	TGAACCCTGACCATTACC
E-cadherin	TACACTGCCCAGGAGCCAGA	TGGCACCAGTGTCCGGATTA
GDH	GGGATTCTAACTACCACTTGCTC	TCTCAATGGCATTAACATAGGC
GAPDH	GCACCGTCAAGGCTGAGAAC	TGGTGAAGACGCCAGTGGA

Table S2: CHIP primers used in this study

Promoter Primers	*Sequence*	
	Fwd	*Rev*
c-Myc	TTGCTGGGTTATTTTAATCAT	ACTGTTTGACAAACCGCATCC
GAPDH	TACTAGCGGTTTTACGGGC	TCGAACAGGAGGAGCAGAGAGCGA

Table S3: shRNA sequences used in this study

Genes	*shRNA sequence*
GRP78-1	5’-CCGGAGATTCAGCAACTGGTTAAAGCTCGAGCTTTAACCAGTTGCTGAATCTTTTTTG-3’
GRP78-2	5’-CCGGGAGCGCATTGATACTAGAAATCTCGAGATTTCTAGTATCAATGCGCTCTTTTTG-3’
c-Myc-1	5’-CCGGCAGTTGAAACACAAACTTGAACTCGAGTTCAAGTTTGTGTTTCAACTGTTTTTG-3’
c-Myc-2	5’-CCGGCCTGAGACAGATCAGCAACAACTCGAGTTGTTGCTGATCTGTCTCAGGTTTTTG-3’
Control	5’-CCGGCCTAAGGTTAAGTCGCCCTCGCTCGAGCGAGGGCGACTTAACCTTAGGTTTTTG-3’
